# Activating transcription factor-3 (ATF3) functions as a tumor suppressor in colon cancer and is up-regulated upon heat-shock protein 90 (Hsp90) inhibition

**DOI:** 10.1186/1471-2407-10-668

**Published:** 2010-12-03

**Authors:** Christina Hackl, Sven A Lang, Christian Moser, Akira Mori, Stefan Fichtner-Feigl, Claus Hellerbrand, Wolfgang Dietmeier, Hans J Schlitt, Edward K Geissler, Oliver Stoeltzing

**Affiliations:** 1 Department of Surgery, University of Regensburg Medical Center, Franz-Josef Strauß Allee 11, 93042 Regensburg, Germany; 2 Department of Internal Medicine I, University of Regensburg Medical Center, Franz-Josef Strauß Allee 11, 93042 Regensburg, Germany; 3 Institute of Pathology, University of Regensburg Medical Center, Franz-Josef Strauß Allee 11, 93042 Regensburg, Germany; 4 Departments of Hepatobiliary and Transplantation Surgery, University Medical Center Hamburg-Eppendorf, Martinistr. 52, 20246 Hamburg, Germany

## Abstract

**Background:**

Activating transcription factor-3 (ATF3) is involved in the complex process of cellular stress response. However, its exact role in cancer is discussed controversially because both tumor suppressive and oncogenic effects have been described. Here we followed-up on our previous observation that inhibition of Hsp90 may increase ATF3 expression and sought to determine the role of ATF3 in colon cancer.

**Methods:**

Regulation of ATF3 was determined in cancer cells using signaling inhibitors and a heat-shock protein-90 (Hsp90) antagonist. Human HCT116 cancer cells were stably transfected with an ATF3-shRNA or a luciferase-shRNA expression plasmid and alterations in cell motility were assessed in migration assays. The impact of ATF3 down-regulation on cancer growth and metastasis were investigated in a subcutaneous tumor model, a model of hepatic tumor growth and in a model of peritoneal carcinomatosis. Human colon cancer tissues were analyzed for ATF3 expression.

**Results:**

The results show that therapeutic Hsp90 inhibition substantially up-regulates the expression of ATF3 in various cancer cells, including colon, gastric and pancreatic cancer. This effect was evident both *in vitro *and *in vivo*. RNAi mediated knock-down of ATF3 in HCT116 colon cancer cells significantly increased cancer cell migration *in vitro*. Moreover, in xenogenic mouse models, ATF3 knock-down promoted subcutaneous tumor growth and hepatic metastasis, as well as peritoneal carcinomatosis. Importantly, ATF3 expression was lower in human colon cancer specimens, as compared to corresponding normal surrounding tissues, suggesting that ATF3 may represent a down-regulated tumor suppressor in colon cancer.

**Conclusion:**

In conclusion, ATF3 down-regulation in colon cancer promotes tumor growth and metastasis. Considering that blocking Hsp90 induces ATF3 expression, Hsp90 inhibition may represent a valid strategy to treat metastatic colon cancer by up-regulating this anti-metastatic transcription factor.

## Background

Heat shock protein 90 (Hsp90) targeting has emerged as a valuable strategy for cancer therapy [1,2], because these proteins are being up-regulated in malignant and non-malignant cells types upon exposure to a variety of stressors [3]. At constitutive levels, heat-shock proteins regulate proper folding and stabilization of abundant intracellular proteins, and their stress-associated induction improves cell survival. Hsp90, one of the most studied molecular chaperons, is overexpressed in tumor cells and is essential for the stability and function of a wide range of oncogenic client proteins [4]. These Hsp90 clients comprise kinases such as ERBB2, EGFR, CDK4, RAF, AKT, cMET and BCR-ABL, and transcription factors such as HIF-1α, STAT3, and STAT5 [2,5,6]. Thus, Hsp90 is a promising target for cancer therapy, as demonstrated by the expanding armamentarium of Hsp90 inhibitors and by new clinical studies incorporating the use of these inhibitors [7]. Nevertheless, due to the broad and complex inhibition of multiple signaling pathways affected by Hsp90, the biological effects remain poorly defined and incompletely understood.

We recently demonstrated that therapeutic inhibition of Hsp90 not only elicits antineoplastic efficacy through blocking oncogenic signaling, but also up-regulates certain signaling molecules in human colon carcinoma cell lines. One of these molecules is activating transcription factor-3 (ATF3), which is Hsp90-inhibitor inducible in HCT116, SW620 and HT29 colon cancer cells [8]. Importantly, such protein up-regulation in response to Hsp90 inhibition has thus far only been reported for certain other heat-shock proteins such as HSF1 and Hsp70. This response may counteract the anti-neoplastic potential of Hsp90 inhibitors for the following reasons [9,10]. ATF3 belongs to the ATF/cyclic AMP response element binding (CREB) family of transcription factors and most cells have very weak or absent ATF3 expression under steady-state conditions. A significant increase in ATF3 can be observed when cell-stress is induced [11], making ATF3 an universal „adaptive response gene" [12,13].

Importantly, different roles for ATF3 have been proposed. In normal tissues, ATF3 may promote both apoptosis and cell proliferation [13], while in neoplasms it has been identified as either an oncogene or as tumor suppressor, depending on tumor entity and grade [13-15]. For instance, ATF3 can mediate pro-apoptotic effects in human mammary epithelial cells, whereas in breast cancer cells (MCF10A) it may promote cell survival, motility and invasiveness [15]. Transgenic mice that overexpress ATF3 in basal epithelial cells develop epidermal hyperplasia, dysplastic lesions and oral squamous cell carcinoma [16]. Also in favor of oncogenicity, the tumor suppressor gene Drg-1 mediates its anti-metastatic properties through ATF3 down-regulation in prostate cancer [17].

In colon cancer, the effects of ATF3 expression are particularly perplexing. In one respect, ATF3 was shown to be overexpressed in human colon cancer specimens and appears to promote tumor growth and migration in an experimental HT29 colon cancer model [18,19]. In another respect, ATF3 has been described to mediate anti-neoplastic and anti-invasive effects of non-steroidal anti-inflammatory drugs (i.e. COX-2 inhibitors) in colorectal cancer [14]. In the present study, we sought to clarify ATF3 regulation and its role in human colon cancer using xenogenic mouse models. We hypothesized that Hsp90 inhibitor-mediated induction of ATF3 expression does not counteract the anti-neoplastic and anti-metastatic potential of Hsp90 targeting agents.

## Methods

### Cell culture

The human colorectal cancer cell lines HCT116, SW620 and HT29 were obtained from the American Type Culture Collection (Manassas, VA). The human gastric cancer cell line TMK-1 was obtained from Eiichi Tahara (University of Hiroshima, Hiroshima, Japan). The metastatic human pancreatic cancer cell line L3.6pl was kindly provided by Dr. I.J. Fidler (The University of Texas, MD Anderson Cancer Center, Houston, TX). HCT116 and SW620 cells were cultured in RPMI 1640, whereas TMK-1, HT29 and L3.6pl were grown in DMEM supplemented with 20% FCS (HCT116 and SW620), 15% FCS (L3.6pl), or 10% FCS (HT29, TMK-1). All *in vitro *experiments were performed at 60 - 70% cell density to reduce effects of confluence. Cell growth rates of transfected cells were assessed by MTT assays, as previously described [20].

### Stable transfection

HCT116 cells were stable transfected with either an ATF3-shRNA (TIB Molbiol; Berlin, Germany) or a luciferase-shRNA (Luc-shRNA) expression plasmid (TIB Molbiol; Berlin, Germany) by using the Lipofectamine transfection reagent (Invitrogen; Karlsruhe, Germany). Cells were grown and expanded in selective medium containing neomycin (G418, Sigma Aldrich, Deisenhofen, Germany). Successful transfection was verified by Western blotting and semi-quantitative PCR for ATF3.

### Reagents and antibodies

The water-soluble Hsp90 inhibitor 17-(dimethylaminoethylamino)-17-demethoxy-geldanamycin (17-DMAG) was purchased from Invivogen (Cayla-Invivogen) and was applied as previously published [8]. Antibodies against ATF3 and anti-β-actin were obtained from Santa Cruz Biotechnology (Santa Cruz, CA). β-actin served as a loading control in Western blotting.

### Western blot analysis

Protein was extracted from whole-cell lysates with RIPA buffer as described before and 50-μg protein samples were subjected to Western blotting on a denaturing 10% sodium dodecyl sulfate-polyacrylamide gel [20]. Membranes were probed for ATF3 and β-actin. For induction of ATF3 *in vitro*, the Hsp90 inhibitor 17-DMAG (100 ng/ml) was added to cell cultures for indicated times and ATF3 protein analysis was performed thereafter. Expression of ATF3 in 17-DMAG treated tumors was similarly determined by lysis of snap frozen tumor tissues and subsequent Western blotting, as described [20].

### Real-time PCR

Real-time PCR was performed as we have previously described [21]. Primer pairs were as follows: ATF3 forward 5-'ctgcagaaagagtcggag-3' and reverse 5'-tgagcccggacaatacac-3'; VEGF-A forward 5'-gcacccatggcagaaggaggag-3' and reverse 5'-agcccccgcatcgcatcag-3'; HIF-1α forward taccatgccccagattcaggat and reverse tcagtggtggcagtggtagtgg; GLUT-1 forward 5'-aactcttcagccagggtccac-3' and reverse 5'-cacagtgaagatgatgaagac-3'. Real-time PCR was done using the LightCycler system and Roche fast-Start Light Cycler-Master Hybridization Probes master mix (Roche Diagnostics) [20].

### Migration Assays

Migration assays were performed using modified Boyden chambers, as described elsewhere [21]. Briefly, 10^5 ^cells were resuspended in 1% FCS medium and seeded into 8-μm filter pores inserts (Becton Dickinson Bioscience). 10% FCS-enriched medium ± 17-DMAG (100 nM) served as chemoattractant. After incubation, migrated cells were stained (Diff-Quick reagent, Dade Behring) and counted in four random fields.

### Animal models

Eight-week-old male nude mice (Charles River, Sulzfeld, Germany) were used. Experiments were approved by the Institutional Animal Care and Use Committee of the University of Regensburg and the regional authorities (Regierung der Oberpfalz, reference number HIF1_2004) and in accordance to the "Guidelines for the Welfare of Animals in Experimental Neoplasia" published by The United Kingdom Coordinating Committee on Cancer Research. In experiments, animals were weighed daily and monitored for weight-loss and other signs of distress.

#### Tumour models

(1) One-million human cancer cells (TMK-1, L3.6pl) were implanted into the subcutis of nude mice, as described [8]. After implantation, tumors were allowed to grow to a volume of 400 mm^3 ^until treatment with either the Hsp90 inhibitor 17-DMAG (3 × 25 mg/kg/week; i.p.), or PBS (control) was started. This dose has proven antineoplastic potential in previous models [8,20]. Tumors were harvested after 14 days of treatment to determine ATF3 protein expression (n = 3 per group).

(2) One-million ATF3-shRNA, or Luc-shRNA transfected HCT116 human colorectal cancer cells were injected into the subcutis of nude mice (n = 9-10 per group). Tumor diameters were measured every other day, and volumes calculated using the estimation: width^2 ^× length × 0.5.

(3) One-million ATF3-shRNA or Luc-shRNA transfected HCT116 cells were injected into the right lower liver lobe of mice to determine hepatic growth, as previously described [22]. Animals were monitored daily and sacrificed on day 28 (n = 9-10/group). Following necropsy, liver weight was measured and all tumor nodules counted and weighed.

(4) For testing peritoneal carcinomatosis, stable transfected HCT116 cells (3 × 10^6^) were implanted into the abdominal cavity by intraperitoneal injection, as previously described [23]. Mice were monitored for 28 days and sacrificed; animals were evaluated for the presence of ascites and tumor nodules were counted.

### Immunohistochemical analysis

Cryosections (7 μm) and paraffin-embedded sections (5 μm) were cut from xenograft tumors for immunohistochemical analyses. CD31-positive vessel area was analyzed by converting images to grey scale and setting a consistent threshold for all slides, as described [20,24]. Vessel area is expressed as pixels per high-power field [20].

### Human tissues

For the analysis of ATF3 mRNA expression, snap frozen tissue samples of primary human colon carcinomas (*n *= 5) and corresponding non-neoplastic colon tissues (*n *= 5) were obtained from the anonymized tumor tissue bank of the Department of Pathology (University of Regensburg), as approved by clinical ethics committee [25]. Tumor characteristics were as follows: #1 sigmoid colon: pT3, L0, V0, pN0, R0; #2 cecum: pT4, pN2 (5/12), M1 (per), G2, R1, L1, V1; #3 sigmoid colon: pT3, pN0 (0/15), G2, R0, L0, V0; #4 cecum: pT3, pN2 (4/24), G3, L1, V0, R0; #5 sigmoid colon: pT3, pN2, G2, R0, L1, V0. Patients did not receive neoadjuvant therapy or chemotherapy before surgery.

### Statistical Analyses

Results from *in vivo *experiments were analyzed for significant outliers using Grubb's test http://www.graphpad.com. Tumor-associated variables in *in vivo *experiments were tested for statistical significance using the Mann-Whitney *U *test for non-parametric data. The two-sided Student *t *test was applied for analysis of *in vitro *data. All results are expressed as the mean ± SEM.

## Results

### Regulation and expression of ATF3 in cancer cells

**Figure 1:**
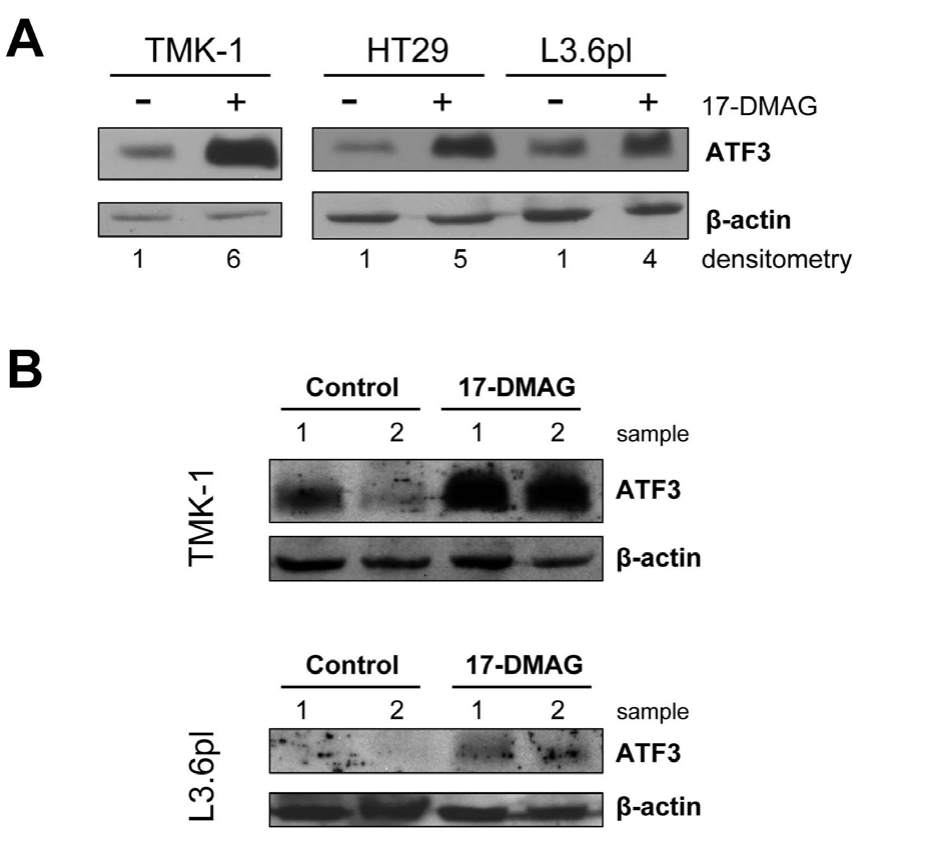
**Induction of ATF3 by Hsp90 inhibition in colon cancer *in vitro *and *in vivo***. Western blot analysis was performed to determine alterations in ATF3 expression upon inhibition of Hsp90 with 17-DMAG. A) Treatment of human gastric (TMK-1), colon (HT29), or pancreatic (L3.6pl) cancer cells with 17-DMAG (100 nM, 16 h) markedly up-regulated ATF3 *in vitro*. B) Similarly, in subcutaneous tumor models of gastric (TMK-1), or pancreatic (L3.6pl) cancer, treatment with the Hsp90 inhibitor 17-DMAG (3 × 25 mg/kg/week) up-regulated ATF3 in protein extracts from excised tumors after 14 days.

We previously observed that treatment of HCT116 and SW620 colon cancer cells with an Hsp90 inhibitor (17-DMAG) substantially up-regulates constitutive ATF3 expression [8]. The biological effects of Hsp90 inhibitor-mediated induction of ATF3 are currently not known. To further validate these results, we investigated whether blocking Hsp90 also leads to ATF3 up-regulation in other human cancer cell types. Indeed we found that blocking Hsp90 induces ATF3 protein expression in human gastric (TMK-1), colon (HT29, HCT116, SW620), and pancreatic (L3.6pl) cancer cell lines (Figure 1A and [8]). These results were validated *in vivo *using a model of subcutaneously implanted gastric (TMK-1), or pancreatic (L3.6pl) cancer cells where Hsp90 inhibitor treatment markedly induced ATF3 expression in respective tumors (Figure 1B).

**Figure 2:**
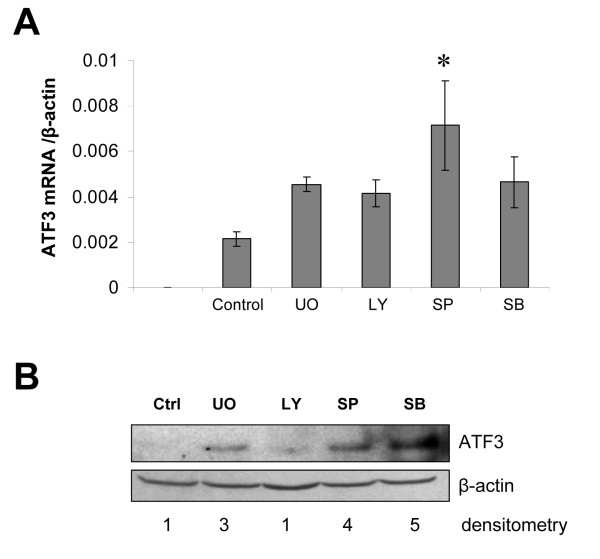
**Identification of signaling pathways for regulating ATF3**. MAPK/Erk (UO), PI-3K/Akt (LY), SAPK (SP), or p38 (SB) inhibitors were used to identify signaling pathways involved in up-regulation of ATF3 in HCT116 colon cancer cells. A) Real-time PCR for ATF3 was performed after 1 hour incubation with indicated signaling inhibitors. Inhibition of SAPK (SP) significantly up-regulated ATF3 mRNA expression (P < 0.05). B) On a protein level, after 20 hours incubation with inhibitors, MAPK/Erk, SAPK and p38 pathways appeared to be involved in mediating ATF3 up-regulation. Bars: mean ± SEM

Since blocking Hsp90 interferes with multiple cell signaling pathways, including MAPK/Erk, PI-3K/Akt, p38 and SAPK, we used in HCT116 cell line selective signaling inhibitors to determine the predominant signaling pathway involved in this Hsp90-inhibitor mediated ATF3 up-regulation (Figure 2). Inhibition of SAPK (SP600125) most robustly up-regulated ATF3 mRNA expression (Figure 2A). However, we additionally observed on a protein level that inhibition of either MAPK/Erk (UO126), or p38 (SB203580), could also up-regulate ATF3 expression in colon cancer cells (Figure 2B). We conclude from these experiments that ATF3 expression in colon cancer cells is complexly controlled through the interaction of multiple molecular signaling pathways. Because Hsp90 inhibition is known to affect a broad variety of signaling pathways, it is reasonable to conclude that inhibitors such as 17-DMAG overall lead to a net-gain in ATF3 expression.

### Effects of down-regulating ATF3 in colon cancer cells

**Figure 3:**
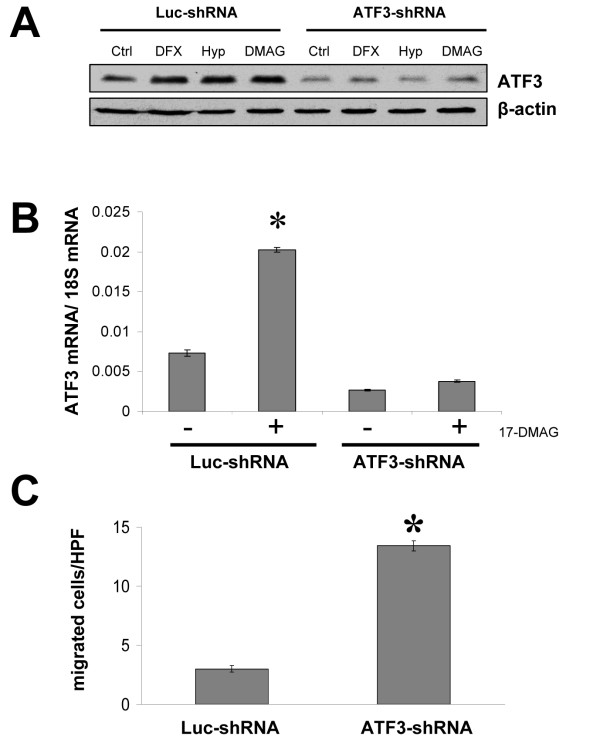
**Effect of ATF3 down-regulation *in vitro***. Human colon cancer cells (HCT116) were stably transfected with either an ATF3-shRNA, or Luc-shRNA (control) vector, for elucidating the functional role of AFT3. A) Western blot analysis showed a constitutive, hypoxia-inducible (1% O_2 _or DFX), and Hsp90-inhibitor (17-DMAG)-mediated ATF3 expression, which was substantially diminished after transfection with the ATF3-shRNA plasmid. B) Stable transfection with the ATF3-shRNA plasmid lowered ATF3 mRNA in HCT116 cells and abrogated the Hsp90 inhibitor-mediated induction of ATF3. C) Down-regulation of ATF3 significantly increased the pro-migration properties of HCT116 cells (*P < 0.05). Bars: mean ± SEM

In view of the fact that ATF3 is stress-inducible and continuously detectable in colon cancer cells, we used an shRNA approach for specifically targeting ATF3 in HCT116 colon cancer cells, with the intention to determine the biological effects of a further ATF3 down-regulation in this cancer entity. Successful stable transfection with an ATF3-shRNA plasmid was verified by Western blotting and real-time PCR (Figure 3A, B). Importantly, down-regulation of ATF3 markedly increased the migration ability of colon cancer cells *in vitro *(Figure 3C). Together, these *in vitro *experiments indicate that ATF3 down-regulation harbors the potential to increase the metastatic potential of colon cancer cells.

### Impact of ATF3 down-regulation on tumor growth *in vivo*

**Figure 4:**
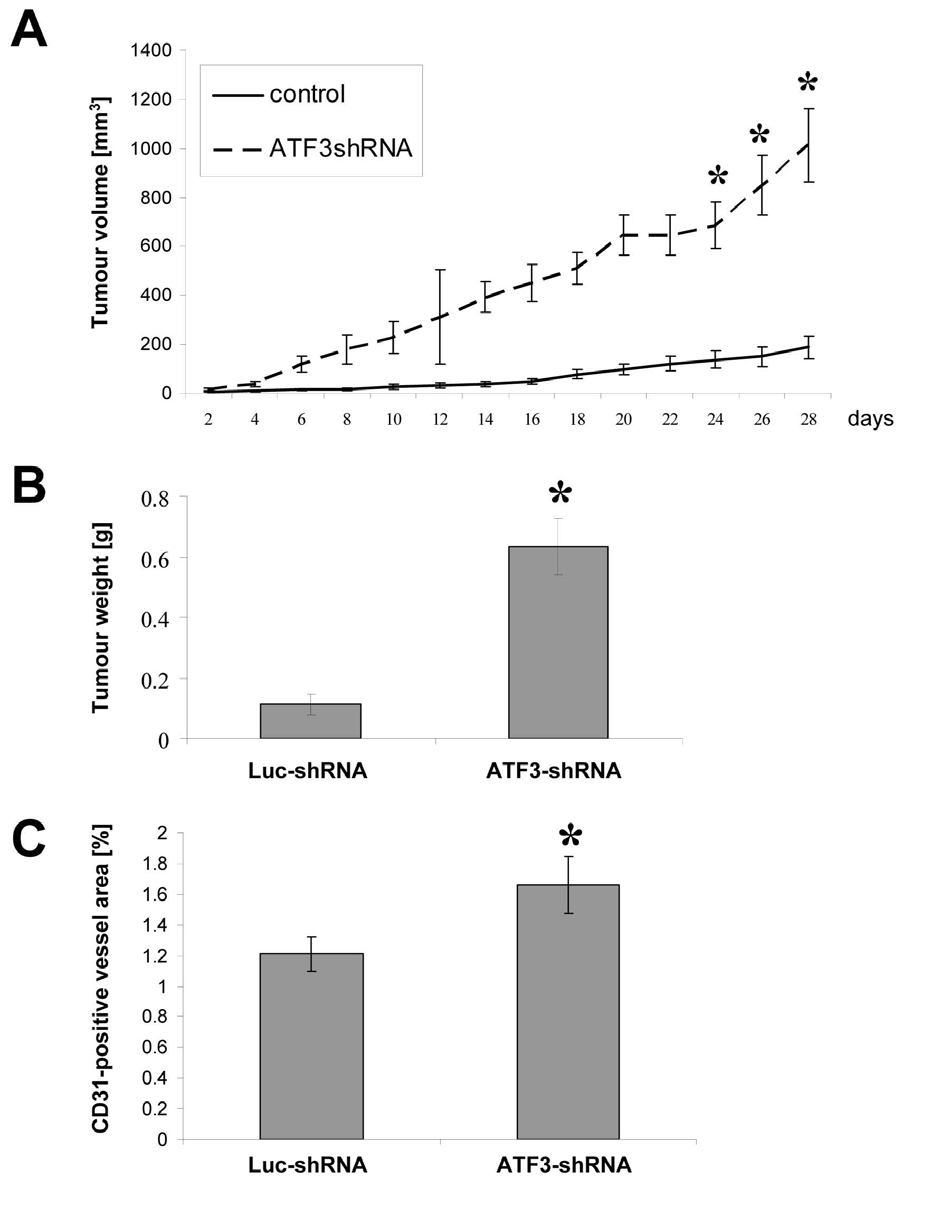
**Impact of ATF3 down-regulation on subcutaneous tumor growth *in vivo***. The effects of ATF3 down-regulation on tumor growth were investigated in a subcutaneous tumor model. A) ATF3-shRNA-transfected HCT116 cells showed an accelerated *in vivo *growth rate, as compared to Luc-shRNA HCT116 cells (*P < 0.01). B) Changes in tumor growth rate were also reflected by final tumor weights at day 28 (*P < 0.01). C) CD31-staining demonstrated increased vascularisation in tumors of the ATF3-shRNA-transfected group, as compared to controls (*P < 0.05). Bars: mean ± SEM

The effects of diminished ATF3 expression on tumor growth *in vivo *were first investigated in a subcutaneous tumor model using HCT116 cells. The results show that down-regulation of ATF3 by ATF3-shRNA leads to an increased tumor growth rate, as compared to Luc-shRNA transfected control cells (Figure 4A, B). Importantly, *in vitro *growth rates of Luc-shRNA and ATF3-shRNA transfected HCT116 cells were statistically not different (data not shown). These *in vivo *results were confirmed by using one additional ATF3-shRNA transfected HCT116 clone (data not shown). Moreover, tumors from mice in the ATF3-shRNA group showed higher vascularisation in terms of an increased CD31-positve vessel area (Figure 4C). We conclude from these experiments that ATF3 functions as a tumor suppressor and growth-inhibitory factor in HCT116 colon cancer.

### Impact of ATF3 down-regulation on colon cancer metastasis *in vivo*

**Figure 5:**
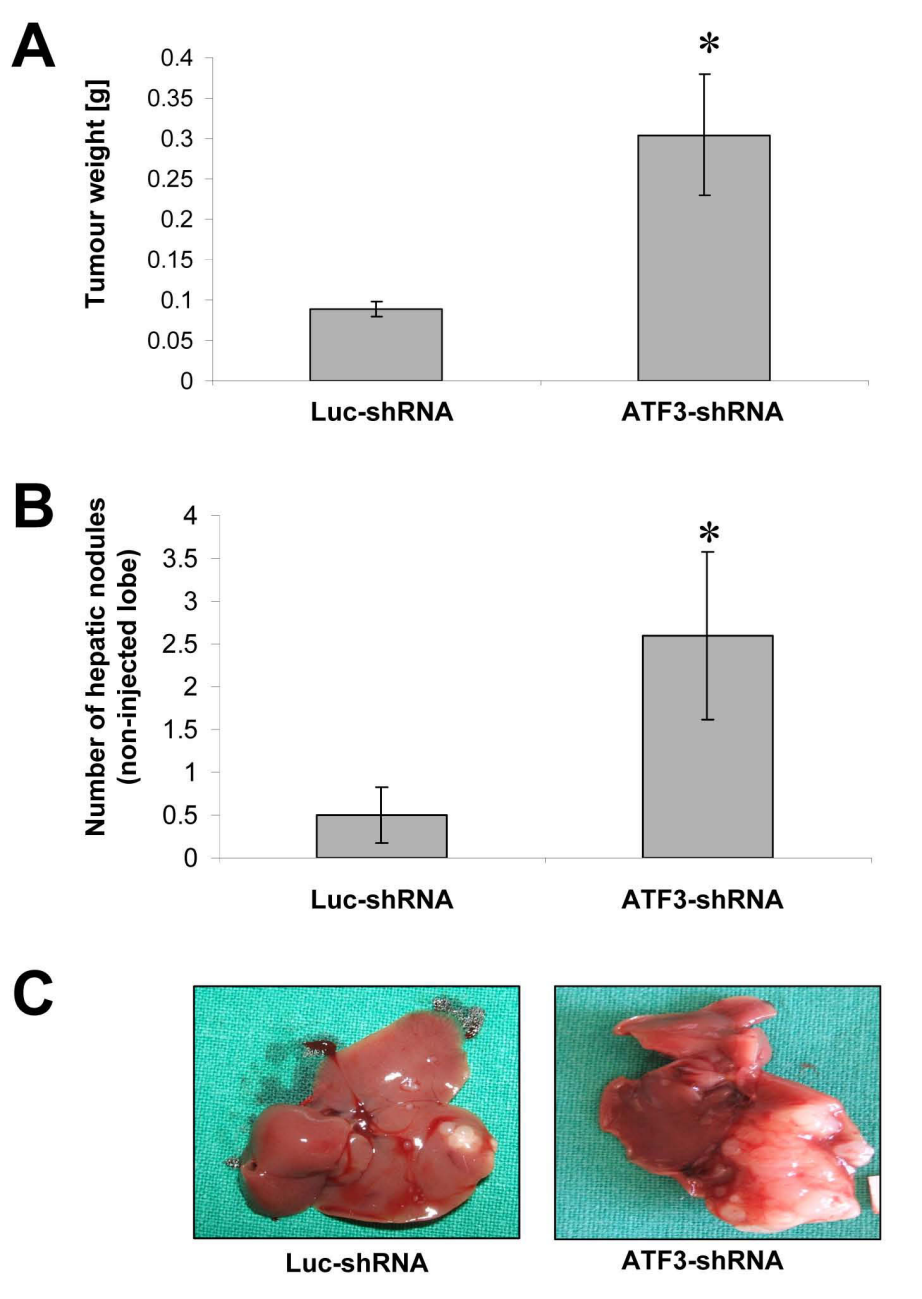
**Effect of ATF3 down-regulation on hepatic tumor growth *in vivo***. A hepatic tumor cell implantation model was used to determine the effects of ATF3 down-regulation on hepatic tumor growth and metastasis of HCT116 cells. A) Animals in the ATF3-shRNA group had a markedly greater hepatic tumor burden, as determined by measuring the weight of excised tumors (*P < 0.05). B) The occurrence of hepatic nodules in liver lobes other than the injected lobe was significantly greater in animals of the ATF3-shRNA group (*P < 0.05). C) Representative images of hepatic tumor burden are shown. Bars: mean ± SEM

**Figure 6:**
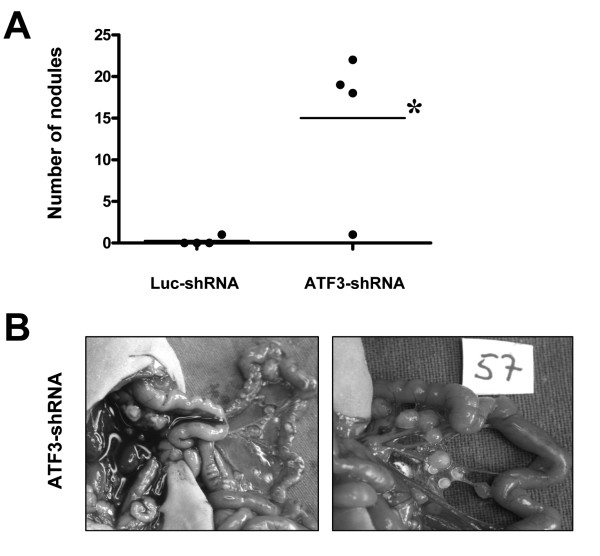
**Effect of ATF3 down-regulation on peritoneal tumor growth and dissemination *in vivo***. Transfected human colon cancer cells (HCT116) were implanted into the peritoneal cavity. A) Down-regulation of ATF3 promoted peritoneal carcinomatosis formation, as determined by counting peritoneal nodules (*P < 0.05). B) Representative images of peritoneal dissemination in the ATF3-shRNA group.

We next tested the effects of inhibited ATF3 expression on tumor metastasis *in vivo *in a model of hepatic tumor growth and in a model of peritoneal carcinomatosis. ATF3 silencing in HCT116 led to a substantial increase in hepatic tumor burden, as compared to Luc-shRNA transfected controls (Figure 5A, B). Furthermore, animals in the ATF3-shRNA group developed significantly more hepatic tumor nodules in liver lobes that had not been injected with tumor cells (Figure 5C). Similarly, in the peritoneal carcinomatosis model, animals in the ATF3-shRNA group developed multiple peritoneal nodules and 2/4 (50%) animals had detectable ascites (Figure 6A, B). These *in vivo *experiments support the hypothesis that ATF3 functions as a tumor suppressor and anti-metastatic factor in HCT116 colon cancer.

### Expression of ATF3 in human colon cancer specimens

**Figure 7:**
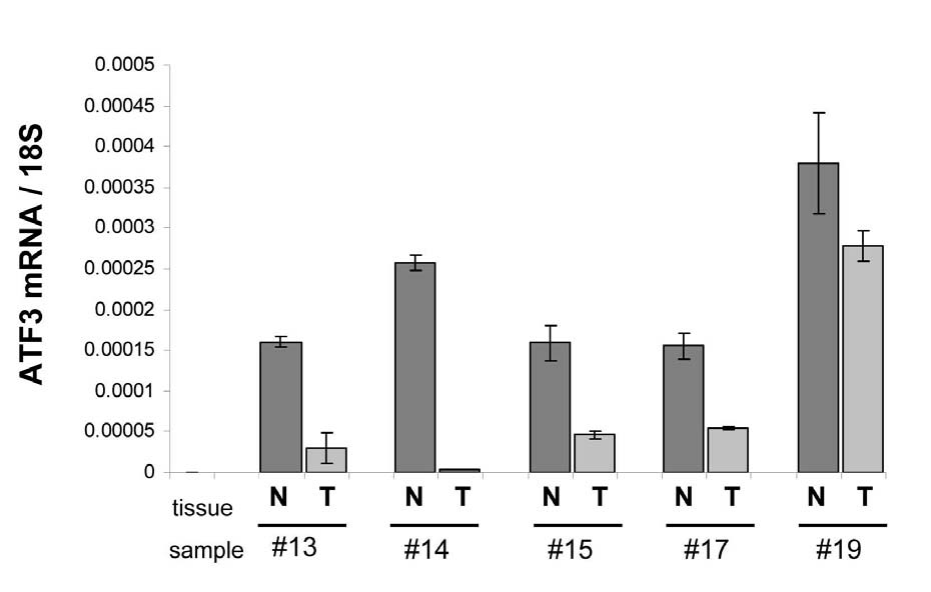
**Expression of ATF3 in human colon cancer**. Corresponding colon cancer and non-tumorous tissues of patients with surgically resected colon cancer (T3-T4) were analyzed by PCR for the expression of ATF3. The expression of ATF3mRNA was markedly reduced in colon cancer specimens. Bars: mean ± SEM

Since studies report conflicting results regarding the role and expression of ATF3 in colorectal cancers, we determined ATF3 mRNA expression in human colon cancer specimens. These results show that ATF3 is consistently expressed at exceptionally low levels in colon cancer tissues, as compared to corresponding normal tissues (Figure 7). We conclude that ATF3 is likely to be down-regulated in colon cancers, hence supporting the rationale of therapeutically inducing ATF3 expression in this cancer entity.

## Discussion

Our recent observation that Hsp90 inhibition induces ATF3 in cancer cells and the lack of clarity regarding the biological effect of this transcription factor in oncology pressed our aim to define the role of ATF3 in colon cancer. We now have confirmed that blocking Hsp90 does indeed induce ATF3 in various cancer derived cell lines, including colon (HT29, HCT116), gastric (TMK1), and pancreatic (L3.6pl) cancer derived cells. Furthermore, this study is the first to demonstrate that loss of ATF3 via shRNA-mediated down-regulation increases the migration properties of HCT116 colon cancer cells *in vitro *and promotes tumor growth and metastasis *in vivo*. Hence, results from this study suggest that ATF3 functions as a tumor suppressor and anti-metastatic factor in HCT116 colon cancer, which is therapeutically inducible by blocking Hsp90.

Recent publications have demonstrated a dichotomous role of ATF3. Depending on the cell type and malignancy, ATF3 can mediate either proliferative and pro-migration properties, or anti-proliferative and pro-apoptotic effects [26-29]. For instance, Yin and co-workers have demonstrated in *in vitro *experiments that ATF3 induces apoptosis in non-malignant mammary epithelial cells, but reduces apoptosis and enhances motility in breast cancer cells, suggesting an oncogenic role of ATF3 in breast cancer [15]. In colon cancer, down-regulating ATF3 in HT29 colon cancer cells with antisense oligonucleotides apparently diminished entopic tumor growth and metastasis in mice [19]. In contrast, we could show that in HCT116 colon cancer, loss of ATF3 function does result in a higher pro-migration capacity *in vitro *and an accelerated tumor growth with increased metastasis *in vivo*. One explanation of this discrepancy might be the different genetic background of HT29 and HCT116 colon cancer cells. While HCT116 harbors mutant KRAS, HT29 colon cancer cells are wildtype for KRAS but harbor mutant BRAF. Recent publications have shown that the KRAS and BRAF mutation status of colon cancer cells influence the expression rates of multiple proliferative as well as apoptotic signaling intermediates (Kikuchi et al, Cancer Res 2009, Oliveira et al, Oncogene 2003, Seruca et al, 2009), including HIF1α signaling and the MAPK/Erk and PI3K/Akt pathways which we identified as interacting with ATF3 (Figure 2). Furthermore, EGFR-targeting agents are clinically effective in the treatment of KRAS and BRAF wildtype tumors, whereas no clinical benefit could be proven for KRAS or BRAF mutant tumors (Lievre et al, Oncogene 2010). Thus, drug-induced overexpression of ATF3 may have beneficial effects in only a subset of colon cancer cells. This important result will be further addressed in future experiments, where loss of ATF3 function as well as ATF3-overexpression will be investigated in colon cancer cells with different genetic background.

In line with our findings in HCT116 colon cancer, tumor suppressive properties of ATF3 were suggested in a study by Oh *et al*., describing that ATF3 acts as tumor-inhibiting factor in HeLa cervical cancer cells *in vitro *[30]. Moreover, Lu and co-workers elegantly demonstrated that ATF3 is capable of suppressing a Ras-mediated tumorigenicity of murine fibroblasts (ATF3^-/- ^versus ATF3^+/+ ^fibroblasts) in an *in vitro*, as well as in an *in vivo *model, hence supporting our hypothesis of a tumor suppressive role. In conclusion, these discrepancies mirror the complex role of ATF3 which may not solely depend on the investigated cell line. The biological function of ATF3 *in vivo *may rather highly rely on the microenvironment of a defined tumor entity.

One clinical significance of our findings is that treatment-induced up-regulation of ATF3, as for example via Hsp90-inhibition or COX-2 inhibition, may be beneficial in some tumors for reducing growth and metastasis [8,14]. With respect to COX-2 inhibitors, experimental studies have nicely demonstrated that ATF3 may mediate anti-neoplastic and anti-invasive effects of such non-steroidal anti-inflammatory drugs [14]. In this study, overexpression of ATF3 inhibited invasion to a similar degree as sulindac sulfide treatment and antisense ATF3 increased invasion *in vitro*. This tumor suppressive effect of ATF3 is also supported by their findings, where transfection of cancer cells with a full-length ATF3 vector suppressed tumorigenicity and invasiveness *in vitro *and tumor growth *in vivo *[14]. However, this group was not able to validate in an *in vivo *setting that loss of ATF3 function is conversely associated with increased growth rates and metastasis, hence our study further expands the knowledge on ATF3 function beyond these aspects. We observed an enhanced migration behavior after ATF3 inhibition *in vitro *and hypothesized that loss of ATF3 function may also lead to an increased tumor metastasis *in vivo*, an aspect that has not been comprehensively investigated to date. In subsequent hepatic and peritoneal tumor models, we were able to demonstrate a significant increase in tumor burden, cancer dissemination, and tumorigenicity upon further down-regulating ATF3. Thus, we propose that ATF3 functions as a tumor suppressor and anti-metastatic factor in HCT116 colon cancer. Moreover, in a recent publication, Ameri and colleagues could show that induction of ATF3 in hypoxic conditions, a common feature detectable in solid tumors, is independent of the transcription factor HIF-1α [11]. The factors HIF-1α and ATF3 are both induced by hypoxia and other cellular stressors, and both transcription factors regulate the expression of multiple genes during tumor progression and metastasis [11].

Importantly, and of high clinical relevance, we could show in the current and in one preliminary previous study that ATF3 expression can be induced in cancer cells by Hsp90 inhibition *in vitro *and *in vivo*. Inhibitors to Hsp90 are currently being investigated in a growing number of clinical trials http://www.clinicaltrials.gov. Thus, the present study not only adds an interesting new aspect to the multiple mechanisms of Hsp90-inhibition, but also provides reasonable evidence that an ATF3-induction by Hsp90 inhibition could be favorable for therapy of advanced colon cancer.

## Conclusion

In conclusion, the present study shows that down-regulation of ATF3 enhances both invasive properties and tumor metastasis of HCT116 colon cancer cells *in vivo*. Our data suggest that induction of ATF3 may be valuable for improving therapy of colorectal cancer patients in terms of preventing hepatic and peritoneal metastasis. Furthermore, our study provides evidence that such ATF3-induction can be achieved by Hsp90-inhibition, which is particularly interesting since Hsp90-inhibitors are promising new agents for targeted therapy of advanced colorectal cancer and other malignancies [2].

## Abbreviation list

17-DMAG: 17-(dimethylaminoethylamino)-17-demethoxygeldanamycin; ATF3: activating transcription factor-3; CREB: cyclic AMP response element binding protein; DMEM: Dulbecco's modified Eagle Medium; GLUT-1: glucose-transporter-1; HIF-1α: hypoxia inducible factor-1alpha; HSF1: heat shock transcription factor 1; HSP70: heat shock protein 70; HSP90: heat shock protein 90; MAPK: mitogen-activated protein kinase; MTT-ASSAY: (3-(4,5-Dimethylthiazol-2-yl)-2,5-diphenyltetrazolium bromide tetrazole assay; PBS: phosphate buffered saline; PI-3K: phosphoinositide-3 kinase; SAPK: stress-activated protein kinase.

## Competing interests

The authors declare that they have no competing interests.

## Authors' contributions

CH - performed majority of experiments, adjusted study design and contributed to manuscript preparation; SAL - performed experiments and involved in study design; CM - performed experiments; AM - performed experiments and aided in animal study; SF - performed statistical analyses and helped with manuscript preparation; CH - collaborator for cell culture experiments; WD - provided human tissues from tissue bank and performed analyses of human specimens; HJS - manuscript editing and study refining; EKG - manuscript preparation and animal study supervision; OS - principal investigator, manuscript preparation, study design, animal study supervision. All authors read and approved the final manuscript

## Pre-publication history

The pre-publication history for this paper can be accessed here:

http://www.biomedcentral.com/1471-2407/10/668/prepub
